# An intensive social cognitive program (can do treatment) in people with relapsing remitting multiple sclerosis and low disability: a randomized controlled trial protocol

**DOI:** 10.1186/s12883-016-0593-4

**Published:** 2016-05-28

**Authors:** Peter Joseph Jongen, Marco Heerings, Rob Ruimschotel, Astrid Hussaarts, Silvia Evers, Lotte Duyverman, Joyce Valkenburg-Vissers, Job Cornelissen, Michel Bos, Maarten van Droffelaar, Wim A. Lemmens, Rogier Donders, Anneke van der Zande, Leo H. Visser

**Affiliations:** Department of Community & Occupational Medicine, University Medical Centre Groningen, Antonius Deusinglaan 1, 9713 AV Groningen, The Netherlands; MS4 Research Institute, Ubbergseweg 34, 6522 KJ Nijmegen, The Netherlands; National MS Foundation The Netherlands, Mathenesserlaan 378, 3023 HB Rotterdam, The Netherlands; Medical Psychiatric Centre PsyToBe, Metroplein 50, 3083 BB Rotterdam, The Netherlands; Department of Health Services Research, Maastricht University, Duboisdomein 30, 6229 GT Maastricht, The Netherlands; Fysiotherapie Maaspoort, Maaspoortweg 182, 5235 KB ‘s-Hertogenbosch, The Netherlands; Dansjobs, Vossestaartstraat 8, 1121 BL Landsmeer, The Netherlands; St. Anna Hospital, Bogardeind 2, 5664 EH Geldrop, The Netherlands; Department for Health Evidence, Radboud University Medical Center, P.O. Box 9101, 6500 HB Nijmegen, The Netherlands; St. Elisabeth Hospital, Hilvarenbeekseweg 60, 5022 GC Tilburg, The Netherlands; University of Humanistic Studies, Kromme Nieuwegracht 29, 3512 HD Utrecht, The Netherlands

**Keywords:** Multiple sclerosis, Self-efficacy, Social cognitive, Wellness, Health-related quality of life, Randomized controlled trial, Relapsing remitting, Disability

## Abstract

**Background:**

In people with multiple sclerosis (MS) disabilities and limitations may negatively affect self-efficacy. Lowered self-efficacy has been associated with decreases in health-related quality of life, physical activity and cognitive performance. In an explorative observational study we found that a 3-day intensive social cognitive program (Can Do Treatment [CDT]) with the participation of support partners was followed by substantial increases in self-efficacy control and health-related quality of life 6 months after treatment in those people with MS who had relapsing remitting disease and low disability.

**Methods/Design:**

CDT is a sociologically oriented approach, its goal is to uncover and promote existing capabilities, and the notion “stressor” is the central concept. CDT’s components are plenary group sessions, small group sessions, consultations, a theatre evening, and start of the day with a joint activity. The small group sessions form the actual training. Depending on their individual goals the participants join the training groups ‘Body’, ‘Feeling’ or ‘Life’, to work out their aims and to reduce their stressors. The multidisciplinary team includes a psychiatrist, psychiatric nurse, neurologist, specialized MS nurse, physiotherapist, dance therapist, and a person with MS. To evaluate the (cost)effectiveness of CDT in persons with relapsing remitting MS and low disability we perform a single-centre, randomized controlled trial in 140 patients, with or without support partners. The primary outcome is self-efficacy control. The secondary outcomes are self-efficacy function, health-related quality of life, autonomy and participation, anxiety, depression, cost effectiveness and cost utility. The tertiary outcome is care-related strain to support partners. Outcomes are assessed at baseline and at 1, 3 and 6 months after CDT.

**Discussion:**

This randomized controlled trial will adequately evaluate the clinical and cost effectiveness of a 3-day intensive social cognitive program in people with relapsing remitting MS and low disability, with self-efficacy control as primary outcome.

**Dutch trial registry:**

Application number: 22444

## Background

Multiple sclerosis (MS) is a chronic disease of the central nervous system pathologically characterized by immune-mediated inflammation, demyelination and axonal degeneration. In most people with MS the first symptoms occur between 20 and 45 years of age, and the initial course is characterized by relapses that are followed by complete or incomplete recovery: relapsing-remitting MS (RRMS) [1]. The nature and intensity of symptoms and disabilities are variable and largely unpredictable. MS cannot be cured and despite the use of disease modifying drugs most persons with RRMS progress to the secondary progressive phase (SPMS), for which no treatment is available. Moreover, the response to disease modifying treatment in RRMS varies between persons and is difficult to predict.

Self-efficacy is a psychological concept that refers to the degree in which a person is confident to complete tasks and reach goals in specific situations [2]. It is a core component in social cognitive theory, according to which psychosocial functioning is determined by reciprocal interactions between personal factors, behavior and the environment [3, 4]. In persons with MS disabilities negatively affect independence, and the negative experience of decreasing independence may result in a decrease in self-efficacy. Low self-efficacy is associated with lower health-related quality of life (HRQoL) [5, 6], less psychological adjustment [7], impaired cognition [8, 9] and less physical activity [6]. Moreover, a lowered self-efficacy may cause patients to underestimate their actual potential, as a result of which capabilities that are still present may eventually disappear. Support partners have also to deal with patients’ disabilities and loss of self-efficacy. A more intensive appeal to them often results in an increased mental and physical strain and may eventually weigh on the relation.

A wellness program is a structured intervention focused on achieving wellness in the physical, psychological and spiritual realm [10]. In the U.S.A. the Can Do Program is a concentrated 4-day interdisciplinary educational wellness program for MS patients, that promotes health seeking behaviors, lifestyle empowerment and exercise [11]. The program aims to enable patients the uncovering of existing capabilities [11], and an improvement in self-efficacy and perceived health has indeed been observed in patients who took part in this program [11].

In an explorative observational study we assessed in MS patients the effect of an intense, multidisciplinary, 3-day, social cognitive wellness program (Can Do Treatment [CDT]) with the participation of support partners [12]. It was found that in the RRMS group 6 months after the intervention the self-efficacy control had significantly increased by 25.8 %, the mental HRQoL by 22.3 % and the physical HRQoL by 17.6 %. Moreover, in the low disability (Expanded Disability Status Scale [EDSS] score <4.0) group the increase in self-efficacy control at 6 months was 23.8 % and the mental HRQoL 19.3 %.

### Aims and objectives

The overall aim of the study is to evaluate in a randomized controlled trial the clinical and cost effectiveness of CDT, with or without the participation of support partners, in comparison to no such treatment for people with RRMS and low disability.

The primary objective is to evaluate the effectiveness of CDT compared to no such treatment in increasing self-efficacy control (primary outcome) during 6 months after treatment.

The secondary objectives are to evaluate:

1. the effectiveness of CDT compared to no such treatment in increasing self-efficacy function, autonomy and participation, HRQoL and coping during 6 months after treatment;
2. the effectiveness of CDT compared to no such treatment in decreasing anxiety and depression during 6 months after treatment;
3. the cost effectiveness and cost utility of CDT during 6 months after treatment;

The tertiary objective is to evaluate the effectiveness of CDT compared to no such treatment in decreasing care-related strain in support partners during 6 months after treatment.

## Methods/Design

### Study design and organization

The study is a single-centre, parallel group, randomized, controlled trial to evaluate the clinical and cost effectiveness of CDT, with or without support partners, in increasing self-efficacy control in RRMS patients with low disability. The 3-day CDT is given to groups of 20 persons in Zorghotel Spelderholt, Beekbergen, The Netherlands, a facility especially equipped for the accommodation of people with impaired health. The first group was treated in April 2013. The ratio between the numbers of patients with support partner and without support partners is about 3:4. The CDTs are given from Friday to Sunday.

The control groups receive no CDT and may receive any care or treatments that are deemed necessary by the treating neurologists, MS nurses or other caregivers (‘care as usual’). In the experimental groups and the parallel control groups the baseline assessment is one week before the CDT, and in all groups the duration of follow-up is 6 months with assessments being performed at 1, 3 and 6 months.

The underlying idea of a 3-day social cognitive intervention program was conceived by the National MS Foundation The Netherlands, Rotterdam, The Netherlands (AvdZ), and further developed in collaboration with PsyToBe (RR, LD) and the other team members. The study is initiated, financed and conducted by the National MS Foundation The Netherlands, and the CDTs are organized and managed by this foundation (MvD). The contributions of the psychiatrist and the psychiatric nurse are part of regular care and are covered by the health insurance. The MS4 Research Institute is responsible for the scientific evaluations.

The study is ongoing (recruiting patients).

### Can do treatment

#### Concept

The goal of CDT is to uncover and promote existing capabilities, with the notion “stressor” as central concept. It is primarily a sociologically oriented approach, as it tries to identify stressors that confine patients with MS to their physical, psychological or social roles. To reduce these stressors, CDT is based on the following principles: identification and reduction of existing stressors; client-centeredness; inclusion of support partner (partner or a significant informal caregiver); group sessions; and self-reliance, autonomy, and acceptance as central themes. Accordingly, CDT focuses on the exploration of stressors that confine patients to their disease and their limitations; reduces the relevant stressors; explores and pushes personal boundaries; and establishes new personal boundaries by making optimal use of the existing potential. To place the individual capabilities in a realistic framework, CDT’s central mottos are ‘Can’, ‘Will’, ‘Choose’, ‘Open up to others’, and ‘Do’. CDT’s messages are that by exploring their boundaries patients become more aware of their faculties and that the resulting self-management leads to higher awareness of potentials and a better communication with care professionals.

#### Components

The CDT’s components are large group sessions, small group sessions, consultations (carrousel), a theatre evening, and start of the day with a joint activity (optionally).

In the plenary group sessions participants make optimal use of their existing potentials, learn how to support and encourage other participants, and experiment how to give the required feedback to the multidisciplinary team; in group sessions in which half of the participants take part stressors are identified that have to be addressed most, and realizable individual aims (one or two) are formulated.

The small group sessions form the actual training. Depending on their individual goals the participants sign up for the training groups ‘Body’, ‘Feeling’ or ‘Life’, to work out their aims and to experiment whether they can reduce their stressors. The Body sessions focus on the exploration of the physical capabilities and are coached by a physiotherapist. The Feeling sessions focus on the exploration of the emotional potential and are coached by a psychiatrist and a psychiatric nurse. The Life sessions focus on the exploration of capabilities relating to the daily living with MS and are coached by a neurologist, a registered nurse specialized in MS and a person with MS. In addition, there are relaxation sessions for those who have difficulties to experience their body: the Dance session focuses on body experience and relaxation, and makes participants aware of the relationship between physical sensations and their emotions and feelings, whereas the Physical session focuses on relaxation through physical strain. The choices between the various small group sessions are made independently by the individual participants themselves.

After having identified and formulated in the large group sessions their individual stressors and aims, the participants sign in for one or more group consultations, during which they verify whether their aims are realizable by asking the members of the multidisciplinary team for aim-related medical information.

On the informal theater evening the participants practice to change roles and to show their potentials by openly experimenting. They do their best to perform before each other and the team. The jointly created evening performance increases the cohesion within the group and learns participants to find an equilibrium between consuming and action.

During an optional joint activity (walk in the woods) at the start of the day the participants experiment with physical challenges and with the management of their energy.

#### Multidisciplinary team

The multidisciplinary team includes a psychiatrist, psychiatric nurse, neurologist, specialized MS nurse, physiotherapist, dance therapist, and a person with MS. The team members respect and understand the participants’ individual qualities and differences, and they stimulate, defy and confront them to explore and push their boundaries. Apart from the consultations, the team keeps to coaching, stimulating and activating the participants. By participating in all large group sessions the team members become acquainted with the individual stressors and goals. During the consultations they have a professional and informative role. In the small group sessions every discipline focuses on its own area of interest. During a tip time at the end of each day the team members evaluate the sessions, inform each other on the participants’ progresses and obstacles, discuss whether the participants make optimal use of the opportunities, and monitor to what extent the personal goals are being attained.

### Eligibility criteria

The eligibility criteria for persons with MS are 1) diagnosis RRMS, 2) being diagnosed at least one year ago,3) EDSS score 4.0 or lower, 4) no symptoms suggestive of a relapse, 5) no relapse in the preceding 4 weeks, 6) willing and capable to participate in the investigations, 7) having access to the internet, and 8) having given informed consent.

The eligibility criteria for the support partners are 1) willing and capable to participate in the investigations, 2) having access to the internet, and 3) having given informed consent.

### Recruitment and inclusion

Participants are recruited by the National MS Foundation The Netherlands via publications in their quarterly journal *Nieuwslijn*, postings on the website www.nationaalmsfonds.nl, information on the website of the organisation’s National MS Network (a portal for MS caregivers), information on social media (facebook, twitter, hyves), and presentations during meetings of neurologists, MS nurses and other caregivers. Persons who are interested are requested to contact the National MS Foundation The Netherlands by phone (+105919839) in order to be informed about the various aspects of the CDT and of the study, like objectives and design. If they wish to receive further information, the patient information leaflet and consent form are sent to them by regular mail, and a second phone call is scheduled with an interval of at least one week. During the second contact by phone, the study assistant (AH) checks the eligibility criteria. If the patient meets the criteria, eventual questions are answered. Persons are explicitly informed that participants in the control group have the right to receive the CDT after their study participation has ended. Questions regarding the content of the CDT treatment are answered by phone by the MS nurse (MH) of the study team or by the principal investigator (PJJ). After another reflection period of at least one week, the MS nurse (MH) contacts the person and if he/she is willing to participate the inclusion procedure follows, during which by use of predefined questions the disease course (RRMS) is verified and the disability (EDSS 4.0 or less) is assessed (see below).

### Ethical and privacy aspects

There are no financial incentives to participate. The expected advantages of study participation are the chances of a clinically relevant increase in self-efficacy and in HRQoL about 6 months after treatment. The disadvantages include the intensive character of the CDT, the completion of questionnaires 4 times over a 6 months period, taking about 45 minutes per time, and the 50 % chance of being randomized into the control group. The risks include the possible occurrence of negative emotional moments, negative effect of the confrontation with one’s symptoms, disabilities and limitations, and accidents during the physical training sessions or joint activity. The CDT constitutes an unusual mental and physical pressure and might therefore lead to the temporary occurrence or worsening of MS symptoms, like fatigue, mood alteration, or emotions. The continuous presence of the experienced team guarantees that unwanted changes are rapidly noticed and adequately cared for.

The study data are coded via an automatically generated code of 10 digits. The identity of the participants is not disclosed in publications or study reports. The protocol has been reviewed and approved by the ethical committee *Medisch Ethische Toetsing Onderzoek Patiënten en Proefpersonen* in Tilburg, The Netherlands. *Medische Ethische Toetsing Commissie* (METC) number: M499. NL number: NL4220502812. The study is being performed in agreement with the Declaration of Helsinki (Ethical Principles for Medical Research involving Human Subjects version 2013; 64th World Medical Association General Assembly, Fortaleza, Brazil, October 2013) (www.wma.net) and the *Wet medisch-wetenschappelijk onderzoek met mensen* (www.wetten.overheid.nl/BWBR0009408). Patients are informed that they have the right to discontinue their participation or withdraw their consent at any time and are not obliged to state their reasons. The completion of the questionnaires takes about 45 minutes per scheduled assessment (baseline and at 1, 3 and 6 months) and the assessment of the EDSS by phone takes about 10 to 20 minutes (see below).

### Data acquisition

The data acquisition is web-based. Patients complete the informed consent form online and start participation by sending their confirmation. After having received a personal code they log on to the website of the MS4 Research Institute (www.ms4ri.nl) and choose a username and password. The study is performed using the LimeSurvey software, an open source online application. There was no testing of the MS4 Research Institute’s platform for this study since it was already being used in various research projects. The items of the questionnaires are fixed and the responses are automatically captured. To protect the personal data from unauthorized access various mechanisms are used to comply with European Union regulations concerning online medical data, including the use of a personal username and a strong password, separation in the database of personal information from the answers to the questions, each screen having a username and password protection, Virtual Private Network tunnelling, 256-bits encryption, and the encryption of the participants’ identities via unique 10 digits codes. Automated completeness checks are done before questionnaires can be submitted. The participants see an overview of all questions and answers before submission and they can change the answers before submitting. After submission changes are no longer possible. The help desk (MH) contacts participants by phone in case they do not succeed in completing questionnaires.

### Primary outcome measure

Self-efficacy control is assessed by the Multiple Sclerosis Self-Efficacy Scale (MSSES). The MSSES is an 18-item, psychometrically validated, self-report questionnaire for the assessment of self-efficacy [13]. The MSSES consists of two 9-item subscales of Function and Control. Each item is scored on a Likert-like scale form 10 (very uncertain) to 100 (very certain) and addition of the respective item scores yields the MSSES-Function score and the MSSES-Control score, both ranging from 90 (minimum) to 900 (maximum). The MSSES-Function subscale measures confidence with functional abilities, whereas the MSSES-Control subscale measures confidence with managing symptoms and coping with the demands of illness [13].

### Secondary outcome measures

Self-efficacy function is assessed with the MSSES (see above).

Participation and autonomy is assessed with the Impact on Participation and Autonomy (IPA) questionnaire, a 32-item, psychometrically validated, generic, self-report instrument for the quantification of limitations in participation and autonomy in people with chronic health conditions [14, 15]. The IPA-Limitations subscale assesses perceived limitations in participation and autonomy in relation to 32 different life situations across five subscales: autonomy indoors, family role, autonomy outdoors, social life and relationships, and work and education [14–16]. Items are rated on a 5-point scale from 0 (very good) to 4 (very poor), and a higher score indicates a higher limitation to participation and autonomy. The IPA-Problems subscale examines the extent to which these limitations are experienced as problematic, by assessing nine different areas of participation and autonomy: mobility, self care, activities in and around the house, looking after money, leisure, social life and relationships, paid or voluntary work, education and training, and helping and supporting other people [14–16]. The perceived problems are graded on a 3-point scale ranging from 0 (no problem) to 2 (severe problems), and a higher IPA-Problems score indicates a greater experience of problems [14–16].

HRQoL is assessed by the Multiple Sclerosis Quality of Life 54-Items (MSQoL-54) and the EuroQoL-5 Dimensions-5 Levels (EQ-5D-5L) questionnaires.

The MSQoL-54 is a psychometrically validated, MS-specific, multi-dimensional inventory of patient-centered health status, and consists of the Short Form 36-Items health survey as a generic core measure, supplemented with 18 questions on items relevant to patients with MS in the areas of health distress, sexual function, satisfaction with sexual function, overall quality of life, cognitive function, energy, and pain and social function [17]. The MSQoL-54 contains 52 items distributed into 12 scales, and two single items. A physical and a mental dimension underlie the MSQoL-54: the Physical and Mental domains [17]. Scores for each domain range from 0 to 100, where higher values indicate better HRQoL.

The EQ-5D-5L is a generic, preference-based, self-reported HRQoL instrument that was developed by the EuroQol Group to measure decrements in health [18, 19]. Respondents record their level of problems experienced in five domains of health: mobility, self-care, usual activities, pain or discomfort, and anxiety or depression, indicating whether they are having no problems or slight, moderate, severe or extreme problems in each assessed domain. Based on the combination of responses, respondents are classified into one of 3125 unique EQ-5D-5L health-state profiles. Each health state is converted to a single utility value representing general population preferences. Utility is measured on a cardinal scale anchored at 1 (perfect health) and 0 (absence of life/dead), and valuations less than zero (as low as -0.594), reflecting health states ‘worse than death’, can exist.

Coping styles are assessed with the Utrecht Coping List (UCL) [20]. The UCL consists of 47 items, measuring 7 independent subscales: active problem solving, palliative reaction, avoidance and passive expectancy, seeking social support, passive reaction pattern, expressing emotions, and comforting cognitions [20, 21]. Active problem solving and comforting cognitions are thought to represent active coping styles, while avoidance, passive reaction and expressing emotions are thought to reflect more passive coping styles. Palliative reaction and seeking social support are related to both active and passive coping styles. The scores on the subscales palliative reaction and avoidance range from 8 to 32, and scores on the subscales active problem solving and passive reaction range between 7 and 28. Scores on subscales emotion expression, comforting cognitions and social support can range from 3 to 12, 5 to 20 and from 6 to 24 respectively.

Anxiety and depression are measured by the Hospital Anxiety and Depression Scale (HADS), a psychometrically validated, 14-item, self-report questionnaire for anxiety and depression [22]. The HADS consists of two subscales, one for anxiety and one for depression, each comprising seven questions. Each question scores 0 to 3 points, and a total subscale score of 0 to 7 points indicate no anxiety/depression, 8 to 10 points indicate possible mild to moderate symptoms of anxiety/depression, and 11 to 21 points indicate a probable clinically significant condition of anxiety/depression [22].

### Tertiary outcome measure

The care-related strain to support partners is measured with the Caregiver Strain Index (CSI). The CSI is a caregiver-specific measure that asks whether aspects of the caregivers’ lives, such as sleep, finances and normal routine have been affected by their caring role, and whether this has placed a physical and mental strain upon them [23, 24]. The CSI includes thirteen items that are scored with Yes (1) or No (0) yielding a total score between 0 and 13. A higher score indicates higher care-related strain, and caregivers' self-reports of experiencing situations that conflict with giving help are associated with CSI scores of 7 or higher [21].

### Other baseline data

Disability is measured by use of the EDSS.The EDSS is based on a neurological examination that provides the basis for the assessment of several functional systems that, according to predefined algorithms, contribute to the EDSS score [25].

### Statistical aspects

Based on the differences in MSSES control scores between baseline and at 6 months after CDT found in RRMS patients with EDSS score <4.0 in the explorative observational study [12], we conducted the following power analysis. We assume at 6 months an average of 650 for the experimental group and an average of 560 for the control group (an increase of 10 points compared to the baseline average found in the observational study). Assuming an SD of 200 on the 6 months measurement we would need 79 patients per group in order to obtain a power of 80 %, when using a two-sided t-test with an alpha of 5 %. However as we will use the baseline measurement in the analysis this number has to be multiplied by a design factor equalling 1 – r^2^_baseline, 6 months_. Assuming a correlation of 0.5 (also based on the observational data) this design factor equals 0.75. Therefore, the number of analyzable patients needed to obtain a power of 80 % equals approximately 60 per condition. Taking account of an expected drop-out of 15 %, we will include a total of 140 patients; approximately 60 with support partner and 80 without support partner.

The randomisation is performed by a statistician who is not otherwise associated with the study (Petra Koopmans PhD, Signidat, Groningen, The Netherlands) via a stratified block randomisation with disease duration and gender as blocking factors. The randomisation list was made at the Department for Health Evidence (RD), Radboud university medical center, Nijmegen, The Netherlands.

The primary outcome variable is the MSSES control score after 6 months. This will be analysed using an ANCOVA with the assessment of 6 months as dependent variable and condition and baseline assessment as independent variables For all other variables the 6 months values will be analysed in a similar way. All analyses will be performed according to the intention-to-treat principle. The statistical analyses will be performed at the Department for Health Evidence, Radboud university medical center, Nijmegen, The Netherlands.

### Economic evaluation

The economic evaluation is a combination of a cost effectiveness analysis (CEA) and cost utility analysis (CUA). In addition to the effect assessments the following instruments will be used: 1) a questionnaire on care use and participation, to gain insight in the costs for health care, the patient and his environment, and the costs outside the health care, 2) the EQ-5D-5L to gain insight into the generic quality of life and utilities. Both instruments are applied simultaneously with the outcomes measures of the effect study. To translate care use and participation in costs, the Dutch Manual for Costing in Economic Evaluations (*Nederlandse Handleiding voor Kostenonderzoek*) will be used [26]. The primary outcome measure for the CEA will be the MSSES control score at 6 months, and the primary outcome measure for the CUA will be the Quality Adjusted Life Years (QALYs). The QALY is a measure in which the number of life years is multiplied with a correction factor the quality of those life years, also called utility. The utilities will be calculated by means of the Dutch tariff that is actually being developed for the EQ-5D-5L. To enable a combined assessment of costs and effects the incremental cost effectiveness ratio (ICER) will be calculated, according to the following formula: $\mathrm{ICER}=\frac{\left({\mathrm{Costs}}_{\mathrm{intervention}}-{\mathrm{Costs}}_{\mathrm{control}}\right)}{\left({\mathrm{Effects}}_{\mathrm{intervention}}-{\mathrm{Effects}}_{\mathrm{control}}\right)}$, wherein ‘Costs intervention’ represents the costs during the whole follow up period in the CDT group, ‘Costs control’ the costs during the whole follow up period in the control group (‘care as usual’), ‘Effects intervention’ the effects at the end of the follow up period in the CDT group, and ‘Effects control’ the effects at the end of the follow up period in the control group. To test for eventual uncertainties that may arise in an economic evaluation study, sensitivity analyses will be performed to assess whether different assumptions lead to different ICER outcomes. To gain insight into sample uncertainty bootstrap analysis will be performed. Based on the results of the bootstrap analysis the ICER uncertainty will be represented graphically. The probability that CDT is cost-effective will be represented with a cost effectiveness acceptance curve.

## Discussion

In an exploratory observational study we assessed in people with MS the potential effectiveness and feasibility of an intensive social-cognitive program with the participation of support partners and found that in the RRMS subgroup self-efficacy control, mental HRQoL and physical HRQoL had increased by 24.8 %, 22.3 % and 17.6 %, respectively. Likewise, in persons with low disability (EDSS score 4.0 or less) the self-efficacy control and mental HRQoL had increased with 23.8 % and 19.3 %, respectively. No such changes were seen in the progressive subgroup or in persons with higher disability (EDSS > 4.0). Therefore, we conceived a randomized controlled trial to further evaluate the (cost)effectiveness of CDT in people with RRMS and low disability. Several other observational studies showed beneficial effects of various types of social-cognitive wellness programs in MS [11, 27, 28], but as yet there are no reports of randomized controlled trials [29].

We considered the MSSES control score the main outcome measure, and not the MSSES function or total score, because the pilot study showed an evident difference between the change in the self-efficacy control vs. the absence of change in self-efficacy function (+1.2 %) [12]. Conceptually, the control and functional subscales of the MSSES measure different dimensions of self-efficacy: the control subscale measures confidence with managing symptoms and coping with the demands of illness, whereas the function subscale measures confidence with functional abilities.

Having had a relapse in the preceding 4 weeks is an exclusion criterion, since we considered that being in a suboptimal condition due to clinical disease activity would increase the risk of unwanted side effects, and would also interfere with the efficacy of CDT. Participants with a relapse during the study period are not excluded from the study, as we do not expect that having experienced a relapse in the 6 month study period or experiencing a relapse at the month 6 assessment does substantially affect self-efficacy; self-efficacy is considered a rather stable trait that requires an intense intervention like CDT to be modified. To address this point in the analysis phase, we assess the on-study occurrence of relapses and will perform a stratified secondary analysis to assess an eventual effect of relapses on the primary outcome.

In an extension phase of 6 months duration we will assess the effect of CDT in the longer term. After the patients in the intervention arm of the study have completed the study they are eligible to be followed for another 6 months, and patients in the control arm have the option to receive the CDT and are also eligible to be followed for another 6 months. Thus, we will be able to assess whether the effect of CDT is sustained on the longer term (12 months), and to assess whether in the control group a similar change in self-efficacy control occurs after CDT as in the RCT intervention group.

An important aspect of complex interventions is their feasibility in real life, e.g. in terms of drop outs, logistics and costs. The drop-out percentage during the 3-day intervention in the pilot study was low (7.4 %) [12], which suggests that the drop out in real life conditions may be limited. As to logistics, in the RCT all CDTs are given at one central location in The Netherlands, as was the case in the pilot study. After the RCT the CDTs will also be given at this location, which has been proven to be easy accessible and well-equipped. Similar approaches could be taken in other countries. A practical aspect of CDT’s implementation and embedding in MS care are the costs in relation to the clinical effects; to be able to weigh the benefits against the costs we investigate the cost-effectiveness of CDT.

Finally, it may be conceived that CDT is influenced by specific aspects of the Dutch health care system and could therefore be of less interest to people with MS treated in other countries. It is of note that a comparable treatment, the Can Do Program, is being given to people with MS in the U.S.A. and improvement in self-efficacy and perceived health was observed in patients who took part in this program [11]. Moreover, none of the components of CDT is related to specific aspects of the Dutch health care system or the Dutch culture. Therefore, in terms of content and effectiveness no relevant differences between countries are to be expected. Yet, the implementation of the study results will depend on the incorporation of CDT in the regular care process, which may differ between countries given the differences in health care systems and reimbursement policies.

## Abbreviations

CEA: cost effectiveness analysis
CDT: can do treatment
CSI: caregiver strain index
CUA: cost utility analysis
EDSS: expanded disability status scale
EQ-5D-5L: euro quality of life 5 dimensions 5 levels
HADS: hospital anxiety and depression scale
HRQoL: health-related quality of life
ICER: incremental cost effectiveness ratio
IPA: impact on participation and autonomy
METC: medische ethische toetsing commissie
MS: multiple sclerosis
MSSES: multiple sclerosis self-efficacy scale
MSQoL-54: multiple sclerosis quality of life-54 items
RRMS: relapsing remitting multiple sclerosis
QALY: quality adjusted life year
SPMS: secondary progressive multiple sclerosis
UCL: Utrecht coping list
